# Small molecule inhibitor screen identifies synergistic activity of the bromodomain inhibitor CPI203 and bortezomib in drug resistant myeloma

**DOI:** 10.18632/oncotarget.4214

**Published:** 2015-05-20

**Authors:** Matthew B. Siegel, Selina Qiuying Liu, Monika A. Davare, Stephen E. Spurgeon, Marc M. Loriaux, Brian J. Druker, Emma C. Scott, Jeffrey W. Tyner

**Affiliations:** ^1^ Knight Cancer Institute, Portland, Oregon, USA; ^2^ Department of Pediatrics at Oregon Health and Science University, Portland, Oregon, USA; ^3^ Howard Hughes Medical Institute, Portland, Oregon, USA

**Keywords:** BET, bromodomain, bortezomib, resistance, myeloma

## Abstract

**Purpose:**

Despite significant therapeutic progress in multiple myeloma, drug resistance is uniformly inevitable and new treatments are needed. Our aim was to identify novel, efficacious small-molecule combinations for use in drug resistant multiple myeloma.

**Experimental Design:**

A panel of 116 small molecule inhibitors was used to screen resistant myeloma cell lines for potential therapeutic targets. Agents found to have enhanced activity in the bortezomib or melphalan resistant myeloma cell lines were investigated further in combination. Synergistic combinations of interest were evaluated in primary patient cells.

**Results:**

The overall single-agent drug sensitivity profiles were dramatically different between melphalan and bortezomib resistant cells, however, the bromodomain inhibitor, CPI203, was observed to have enhanced activity in both the bortezomib and melphalan resistant lines compared to their wild-type counterparts. The combination of bortezomib and CPI203 was found to be synergistic in both the bortezomib and melphalan resistant cell lines as well as in a primary multiple myeloma sample from a patient refractory to recent proteasome inhibitor treatment. The CPI203-bortezomib combination led to enhanced apoptosis and anti-proliferative effects. Finally, in contrast to prior reports of synergy between bortezomib and other epigenetic modifying agents, which implicated MYC downregulation or NOXA induction, our analyses suggest that CPI203-bortezomib synergy is independent of these events.

**Conclusion:**

Our preclinical data supports a role for the clinical investigation of the bromodomain inhibitor CPI203 combined with bortezomib or alkylating agents in resistant multiple myeloma.

## INTRODUCTION

Multiple myeloma (MM) is a hematologic malignancy characterized by bone marrow infiltration of neoplastic plasma cells. Despite significant therapeutic progress, MM remains incurable. Currently the most effective treatment strategies for MM rely on combination regimens including novel agents (proteasome inhibitors or immunomodulators) and standard chemotherapies (alkylating agents, steroids, topoisomerase inhibitors). However, therapeutic options become limited over the course of the disease resulting from the development of drug resistance, treatment related toxicity, and intolerance [1]. While the median overall survival for MM at the time of relapse in the era of novel agents is greater than 30 months, a recent study found that once a patient becomes refractory to bortezomib (BTZ), the median overall survival diminishes to only 11 months [2]. Thus, the development of new strategies to overcome the resistance to novel agents is needed.

With a rapidly increasing number of targeted agents available in the preclinical setting, an effective and high throughput approach for identifying active agents followed by rationally designed treatment is desirable. We have developed a panel of molecularly targeted small-molecule inhibitors for direct interrogation of primary leukemia patient samples that collectively informs pathway dependence, and identifies candidate therapeutic options [3]. In our present study, we used a similarly constructed panel of 116 targeted agents to screen MM cells, including BTZ and melphalan resistant lines for the establishment of rationale drug combinations for resistant MM. The compounds comprising this panel were selected using three basic criteria 1) clinical relevance, 2) breadth of coverage of both kinase and non-kinase targets, and 3) knowledge of on- and off-target activities. As a result, this panel of agents exhibits activity against two-thirds of the tyrosine kinome as well as numerous other kinase families including PI3K/AKT, PKC, PKA, IKK, RAF/MEK/ERK, JNK, p38, AMPK, aurora kinases, and cyclin-dependent kinases. In addition, this panel also exhibits coverage of a variety of non-kinase targets including the proteasome, HSP70/90 proteins, the BCL2 and BET families, as well as the WNT/β-catenin, and sonic hedgehog pathways. A bromodomain inhibitor was among the compounds that showed particular promise in the drug resistant cell lines and therefore was the focus of our follow-up investigation.

BET (bromodomain and extra-terminal) family proteins include BRD2, BRD3, BRD4, and BRDT and are the so called “readers of the histone modification code”. Bromodomain (BRD)-containing proteins selectively bind to the acetyl lysine marks placed by histone modifying enzymes (HATs, HDACs) [4]. BET inhibitors are a promising new class of anticancer agents because BET family proteins BRD2/3/4 are chromatin adaptors, functionally linked to pathways important for cellular viability and cancer signaling. In particular, the direct role of BRD4 in regulating transcription has recently been described. BRD4 is an atypical kinase that directly phosphorylates the serine 2 site of the carboxyl-terminal domain of RNA polymerase II, which is required for the recruitment of RNA splicing factors. Therefore, BRD4 has a direct role in regulating transcription [5]. Targeted BET inhibition using (+)-JQ1, a selective BRD4 inhibitor leads to the downregulation of MYC [6-8]. While MYC downregulation has been the focus of most BRD4 inhibitor studies, it has been shown to represent only one of nearly 3000 transcriptional changes induced by (+)-JQ1 [9]. (+)-JQ1 has shown promising activity in multiple preclinical cancer models [6, 7, 10-15], including MM xenograft models [6].

MYC pathway activation can be involved in MM pathogenesis through a variety of mechanisms. Transcriptional profiling identifies MYC pathway activation in up to 67% of MM patient samples [16]. In early MM increased MYC RNA expression can be seen, while later in the disease MYC rearrangement can occur and is associated with aggressive progression involving extramedullary disease [17]. Further, interferon regulatory factor 4 (IRF4), a transcription factor, can become dysregulated and contribute to the activation of MYC independent of the oncogenic transforming mechanism [18].

Report of a myeloma mouse model involving a MYC transgene showed activity with (+)-JQ1 alone and in combination with bortezomib or a deacetylase inhibitor. In particular it was noted that activity with these agents persisted even after the model was serially passaged to induce BTZ resistance [19]. In our present study, we tested CPI203, a readily bioavailable selective BET inhibitor (BRD4 IC50 = 26 nM) with similar biologic activity to (+)-JQ1 [5, 20]. CPI203 has been evaluated in a preclinical BTZ resistant mantle cell lymphoma model associated with increased basal MYC expression. In that setting, CPI203 was found to exert antitumor effects through the downregulation of MYC and IRF4 [8]. Our data further characterizes the activity of BET inhibition using CPI203 in BTZ resistant MM and its synergistic role with BTZ in this setting.

## RESULTS

### Small molecule inhibitor screen confirms known MM targets

Using a panel of 116 targeted agents, we performed cell viability testing on BTZ (ANBL6 BR and 8226.BR) and melphalan (8226/LR5) resistant cell lines, in addition to the well characterized “parent” MM cell lines U266, ANBL6 WT, and RPMI8226 from which the resistant lines were derived.

Concentration of inhibitor required to suppress cell growth and viability by 50% (IC50) was obtained for each individual agent across the various cell lines. In order to prioritize on-target effects across the large panel, we compared the IC50 values from the tested MM cell lines to the pooled results of 151 patient bone marrow aspirate samples tested on the same panel that we have previously published [3]. Table 1 displays these normalized values (referred to as R50). Higher R50 values represent increasing hypersensitivity of the myeloma cells to a particular agent relative to the collective experience of that same agent over our previously published cohort of 151 diverse hematologic malignancy specimens [3]. The small-molecule inhibitors with the top 10 greatest R50 values included three PKC inhibitors, two Aurora kinase inhibitors, two PI3K inhibitors, an AKT inhibitor, a CDK2 inhibitor, and the multi-kinase inhibitor, sorafenib. These hypersensitive pathways identified by our screen are well supported by the work of others who have described these pathways as promising targets in MM [21-29].

**Table 1:** Top 10 R50 values with corresponding IC50 values

R50 (median patient IC50 ÷ median pooled MM cell line IC50)	Median patient IC50 from ref[3] (nM)	Median IC50 of pooled MM cell lines (nM)	Drug name	Target
17.1	6888.0	404.0	PKC-412	PKC
15.5	108.0	7.0	Staurosporin	PKC, wide range of targets
14.1	10000.0	710.6	MLN-8054	Aurora A
12.1	10000.0	824.9	VX-680	Pan-Aurora
6.0	2217.0	368.5	PI-103	PI3K
4.3	1475.0	340.2	AKT IV	AKT
2.3	10000.0	4257.6	Go6976	PKC
1.9	4307.0	2323.7	LY294002	PI3K
1.5	190.0	127.7	BMS-387032	CDK2
1.1	10000.0	8821.6	Sorafenib	VEGFR, PDGFR, RAF

R50 values represent the pooled patient sample median IC50 value divided by the median IC50 value from the myeloma cell lines tested. Therefore, the R50 value represents the fold magnitude increased sensitivity to a drug in the MM cell lines compared to the pooled database control. IC50 data for each drug for each patient specimen used to generate the median patient IC50 values have been previously published [3]. Note that the RPMI 8226 IC50 values used represents a mean of the wild type RPMI 8226 strains that were obtained from 3 separate sources as described in Table 2. IC50 values are listed as nanomolar units. See supplementary Table S2 for the complete table of all agents tested.

### Bortezomib and melphalan resistant cell lines have distinct targeted pathway sensitivity profiles

**Figure 1:**
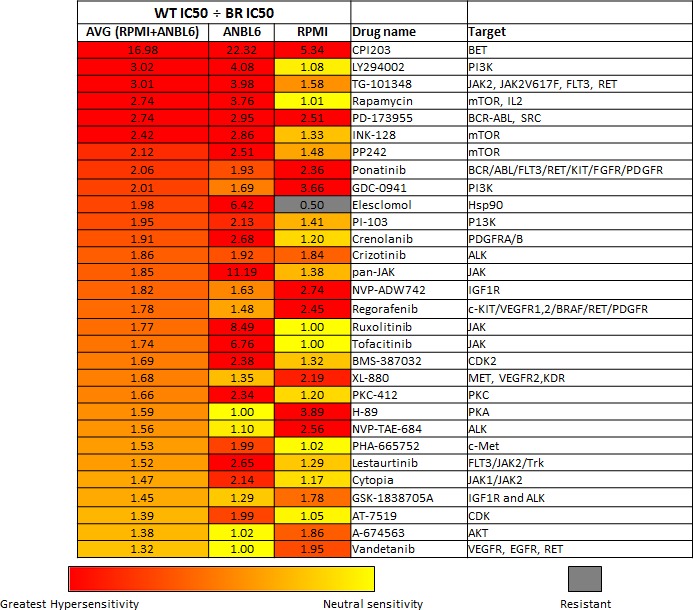
Enhanced drug sensitivities in bortezomib resistant cells The values listed under WT IC50 ÷ BR IC50 represents the fold magnitude increased sensitivity in the BTZ resistant cell lines as compared to their wild type parent cell lines (IC50s for ANBL6 WT ÷ ANBL6 BR, and RPMI 8226 ÷ 8226.BR). These values were obtained by dividing the IC50 values for the wild type MM cell lines (RPMI 8226 and ANBL6 WT) by the corresponding IC50 value of the BTZ resistant cell line. The table is in rank order for the top 30 drugs with the greatest increase in sensitivity in the BR cell lines. Relative hypersensitivity displayed as heatmap as follows: Red indicates greatest hypersensitivity in the BTZ resistant cells relative to its paired BTZ sensitive cell line. Yellow or a numeric value of 1.0 indicates neutral sensitivity (sensitivity in BTZ resistant cells is equivalent to its paired BTZ sensitive cell line). Gray indicates greater resistance in the BTZ resistant cells relative to its paired BTZ sensitive cell line. See supplementary Table S4 for the table of all 116 agents tested.

One can postulate that the biological alterations that occur upon acquiring resistance to one drug may lead to an increased sensitivity to another drug. Given our aim to identify novel combinations for drug resistant disease, we directly compared the differences in the IC50 values between our resistant lines (8226.BR, ANBL6 BR, and 8226/LR5) with their wild type parent lines (RPMI8226 and ANBL6 WT). Dividing the wild type IC50 values by the resistant cell line IC50 values represents the relative fold magnitude increase sensitivity the resistant lines have to a particular agent. Figure 1 and supplementary Table S3 display the drugs with the greatest increased sensitivity in the resistant cell lines (highest IC50 WT line/IC50 resistant line). Interestingly the mTOR, PI3K, JAK, ALK, and IGFR1 inhibitors gained the most sensitivity upon BTZ resistance, while proteasome, aurora kinase, and HER2 inhibitors gained the most efficacy within the acquired melphalan resistance setting. While there was very little overlap in general between the melphalan and BTZ resistance profiles, CPI203 emerged as one of the top drugs in both the melphalan and BTZ resistant settings. CPI203 showed the greatest increase in efficacy in the BTZ resistant cell lines, at nearly 17 fold increased efficacy in the BR setting compared to its WT counterpart. CPI203 also showed increased sensitivity in the melphalan resistant cell line relative to its WT parental line.

### CPI203 and bortezomib are synergistic *in vitro*

**Figure 2:**
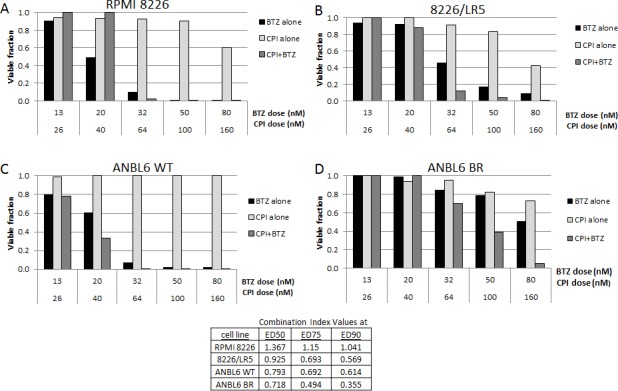
Synergy of CPI203 and BTZ on cell viability assay in cell lines CPI203 synergizes with BTZ in melphalan and BTZ resistant cell lines. Parental **A.**, **C.** and melphalan **B.** and BTZ **D.** resistant cell lines were treated with CPI203 and BTZ as single agents and in combination for 72 hours and relative cell viability was measured using a tetrazolium-based MTS assay. The corresponding table lists the combination indices at ED50, ED75, and ED90.

The activity of CPI203 was of particular interest due to the impressive enhancement of activity in both the BTZ and melphalan resistant cell lines compared to their wild type counterparts. We therefore investigated CPI203 in combination with BTZ. The combination of CPI203-BTZ showed synergy in the MTS cell viability assay in both ANBL6 WT and ANBL6 BR, as well as in 8226/LR5, but not the wild type RPMI8226 based on calculated CI values (Figure 2). The greatest degree of synergistic activity was seen in the BTZ resistant line based on the CI of 0.35 at ED90 drug concentrations. The lack of synergy identified in wild type RPMI8226 is likely due to its exquisite baseline sensitivity to single agent BTZ.

**Figure 3:**
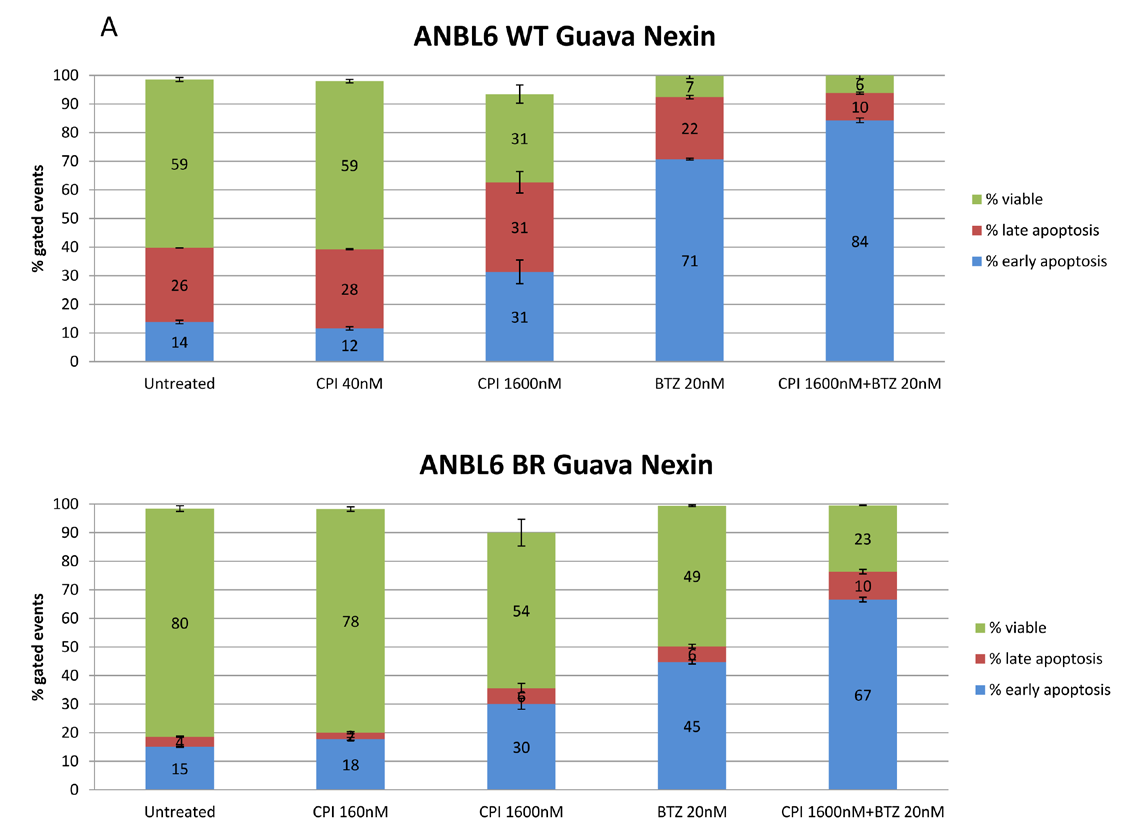

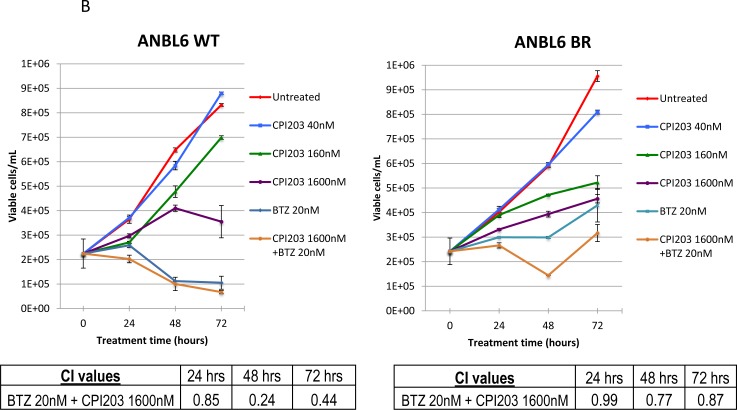

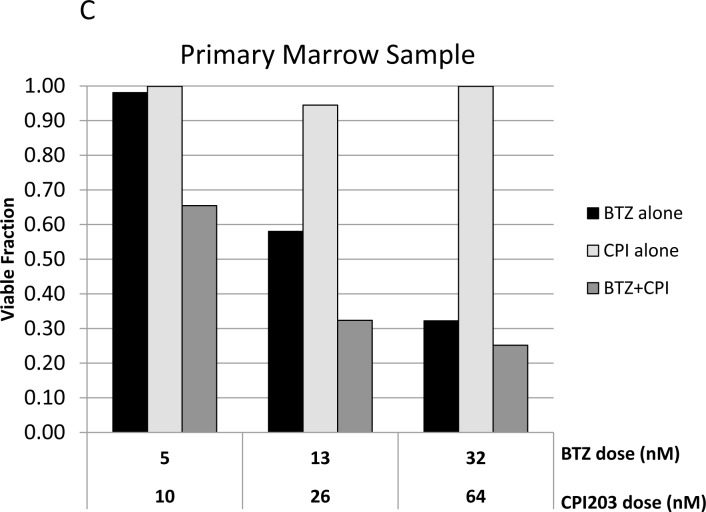
CPI203 and BTZ are synergistic in assays of apoptosis, cell growth, and cell viability in a patient sample **A.** Parental and BTZ-resistant cell lines were treated with CPI203 and BTZ as single agents and in combination for 72 hours and apoptosis was measured using annexin V and 7-AAD staining by flow cytometry. Treatment of ANBL6 WT cells with the combination of 1600nM CPI203 + 20nM of BTZ led to apoptosis in 94% of the cells, compared 40% apoptosis in untreated (no drug) cells (*p* = 0.0017). Likewise in ANBL6 BR cells, 76.8% of the cells treated with the combination of 1600nM CPI203 + 20 nM BTZ were apoptotic vs 19% in untreated cells (*p* = 0.0001). **B.** Parental and BTZ-resistant cell lines were treated with CPI203 and BTZ as single agents and in combination. Viable cell numbers were enumerated by propidium exclusion using a Guava viacount at 0, 24, 48, and 72 hours after treatment. The corresponding table lists the combination indices for various drug dose combinations at each time point. **C.** Primary cells from a bone marrow aspirate obtained from a patient with proteasome refractory MM were exposed to CPI203 and BTZ as single agents and in combination for 48 hours. Relative cell viability was measured using a tetrazolium-based MTS assay. The combination index at ED50 is 0.23 indicating strong synergy.

We next aimed to determine if the inhibitory effect of CPI203 was due to apoptosis or was primarily anti-proliferative. Treatment of ANBL6 WT and ANBL6 BR with various combinations of BTZ and CPI203 for 72 hours induced the accumulation of cells in early apoptosis marked by annexin V+, 7-AAD negative, and to a lesser extent late apoptosis (Annexin V+, 7-AAD+). At low concentrations of CPI203 (40nM in WT line, or 160nM in BR line), there was no enhancement of apoptosis either as single agents or when added to BTZ. At a concentration of 1600nM, CPI203 exerted increased apoptosis as a single agent as well as in combination with BTZ (Figure 3A).

To better understand the effects of the combination on cell growth kinetics, ANBL6 WT and BR cells were exposed to different concentrations of BTZ and CPI203, and serial cell counts were assessed based on guava ViaCount over the course of 72 hours (Figure 3B). BTZ treatment led to a clear reduction in the number of viable cells compared to the starting cell count, while cells treated with single agent CPI203 exerted more of a cytostatic effect indicating that CPI203 may be anti-proliferative at lower doses. Apoptosis was only seen at higher concentrations or when combined with BTZ.

We next examined the effects of CPI203 and BTZ in a primary patient bone marrow sample from a patient with relapsed-refractory MM with 90% marrow involvement, who had previously taken BTZ and progressed on carfilzomib (2^nd^ generation proteasome inhibitor). Cytogenetic analysis of the MM sample showed high risk genetic features including: chromosome 17p deletion, gain of 1q, t(4;14), and monosomy 13. A significant synergistic effect was shown at low nanomolar concentrations of BTZ and CPI203 at 48 hours, even overcoming the cytoprotective effects of the native bone marrow microenvironment (Figure 3C).

### CPI203 and BTZ synergy is independent of MYC downregulation or NOXA induction

**Figure 4:**
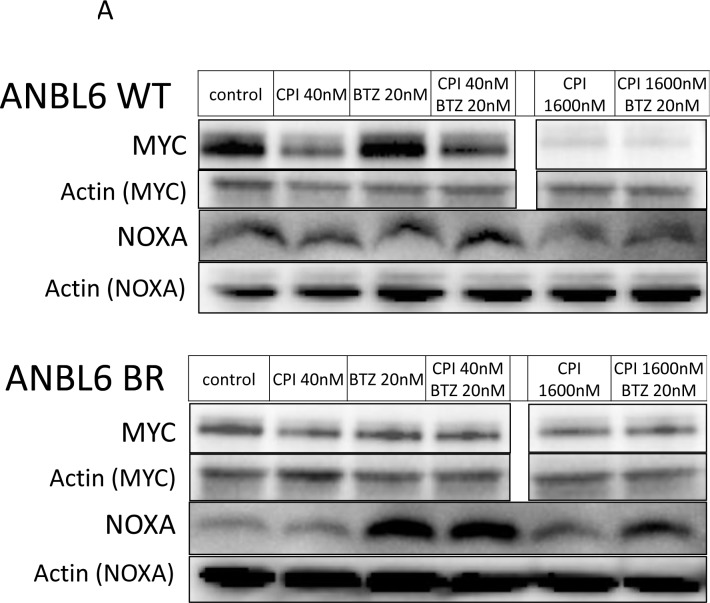

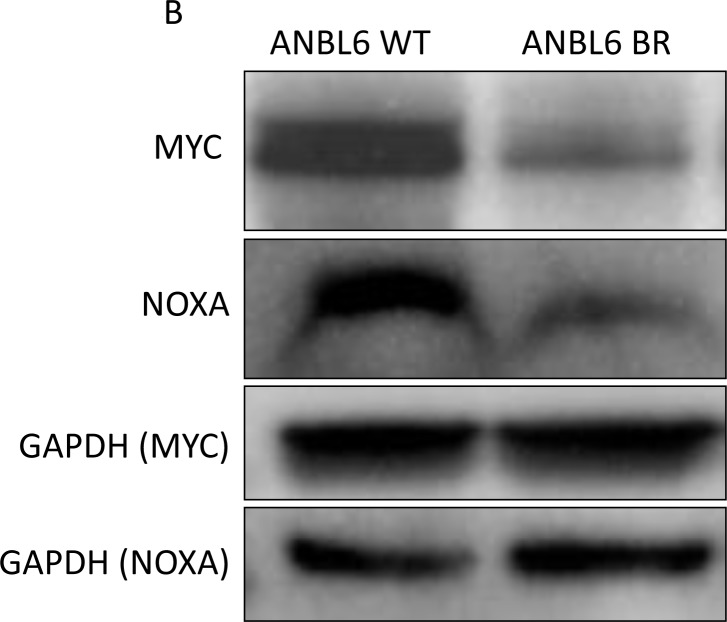
Synergy is independent of change in NOXA and MYC expression **A.** ANBL6 WT and BR cell lines were exposed to CPI203 and BTZ as single agents and in combination and whole cell lysates were subjected to immunoblot analysis using antibodies specific to MYC and NOXA. Note that lanes in horizontal rows were not necessarily contiguous, but were on the same blot thus exposure time for chemiluminescence was identical. **B.** ANBL6 WT and BR cell lysates were subjected to immunoblot using antibodies specific for MYC and NOXA to evaluate basal expression levels.

Studies have illustrated that the effects of proteasome inhibition converge with the induction of NOXA, a proapoptotic protein that triggers cell death through both caspase- and non-caspase-dependent pathways [30]. NOXA has been shown to be under transcriptional regulation by MYC [31]. Given that small molecule BET inhibitors have been shown to reduce the expression of MYC [5-8, 20], we further explored the synergistic mechanism between BTZ and CPI203. Immunoblotting of lysates from ANBL6 WT and ANBL6 BR cells treated with BTZ and CPI203 for NOXA and MYC revealed that single agent BTZ induced the expected dose-dependent rise in NOXA, but did not appear to significantly impact MYC expression levels. Single agent CPI203 led to a dose-dependent decrease in MYC, but had little effect on NOXA expression. When BTZ was combined with CPI203, there was a relative decrease in the induction of NOXA compared to cells treated with BTZ alone. The combination did not appear to have any further impact on MYC levels compared to the effects of CPI203 alone. We also performed immunoblotting of untreated cell lysates to determine if the enhanced sensitivity of ANBL6 BR to CPI203 was related to any differences in basal NOXA or MYC expression (Figure 4). There were relatively reduced basal levels of both MYC and NOXA expression in ANBL6 BR compared to WT.

## DISCUSSION

The panel of small molecule inhibitors we used to screen myeloma cell lines contained many agents with multitarget and overlapping inhibitory properties. This built in redundancy of the panel enabled us to better evaluate the on-target effects of agents targeting multiple nodes of a common signaling pathway. Concordant results between multiple agents of varying chemotype that target the same pathway provide additional validation of the identified candidate pathways. We showed that this screening panel was able to detect well-known MM targets as the “top hits” which provides strong evidence that accuracy is maintained within this high-throughput assay. This panel approach allowed us to rapidly identify many promising targets and individual compounds for further investigation, many of which remain unexplored.

As drug resistance has major treatment and prognostic implications in MM, we focused on further describing CPI203, an agent that showed intriguing effectiveness in drug resistant cell lines on our small-molecule inhibitor screen. The ability of CPI203 monotherapy to suppress growth was enhanced in both the BTZ and melphalan resistant cells compared to their wild type counterparts. Further, the combination of CPI203-BTZ was synergistic in both the BTZ and melphalan resistant cell lines as well as in a primary bone marrow sample from a patient with MM. In BTZ resistant cells, the CPI203-BTZ combination appeared to have antiproliferative effects as well as enhancement of apoptosis. Given that CPI203 is thought to work in part by reducing MYC levels it was surprising to find lower basal MYC expression in the BTZ resistant cell line despite being more sensitive to this drug. Further, the diminished MYC expression in the BTZ resistant cell line is contrary to a prior report that BTZ resistance is associated with increased MYC expression [8]. Moreover, the CPI203-BTZ combination failed to further reduce MYC expression and did not enhance NOXA induction over BTZ alone. Though a 20 nM dose of BTZ did not cause significant apoptosis in the BTZ resistant line, there was strong NOXA induction at this dose, suggesting that these resistant cells are able to survive despite activation of the NOXA mediated apoptotic pathway. While we showed that a synergistic relationship between CPI203 and BTZ exists, the mechanism for the synergy and hypersensitivity of BTZ resistant cells to CPI203 is less clear but appears to be independent of MYC or NOXA alterations.

Just as the mechanism of BTZ efficacy appears to be multifactorial, so does the mechanism for drug resistance. While multiple BTZ resistant cell lines have been evaluated, a unifying mechanism for resistance remains elusive. Reported mechanisms include overexpression or mutations in PSMB5 (the proteasomal target of BTZ), P-glycoprotein expression (efflux pump), microenvironmental upregulation of IL-6, IGF-1, or tumor upregulation of HSP90, PI3K, MYC, and IGF-1 [8, 32-36]. BTZ resistant cell lines are reported to have reduced NOXA and caspase 3 activation in response to BTZ [37, 38]. In ANBL6 BR specifically, it has been shown that increased IGF-1 expression and secretion as well as increased AKT expression likely contributes to resistance. Likewise, it has been reported that inhibitors of the PI3K/mTOR and IGF-1R can act synergistically with BTZ, and may re-sensitize BR cells to BTZ [33]. Our data supports the finding of enhanced sensitivity to PI3K/mTOR pathway inhibitors in the BTZ resistant cell lines, and we also found enhanced sensitivity to IGF-1R inhibition in the 8226.BR cell line (data shown in Figure 1).

Additionally, a recent report suggests that higher MYC expression promotes survival through activation of the PERK/eIF2alpha/ATF4 dependent aggresome pathway, a resistance mechanism known to be important in the setting of proteasome inhibition [39]. Evidence for MYC overexpression leading to enhanced proliferation, extramedullary progression, and possibly BTZ resistance provides the rationale for targeting MYC inhibition in effort to re-sensitize BTZ resistant MM to proteasome inhibition. This is further supported by data published showing activity of a BET inhibitor in a BTZ resistant transgenic MYC activated mouse model [19].

Our data show that treatment with CPI203 sensitized both BTZ resistant and sensitive cells to BTZ despite the CPI203 mediated downregulation of MYC and NOXA expression. The mechanism for the synergistic effect in the BTZ resistance setting is unlike those previously described in the literature. In contrast to previous reports [31, 40] showing a reliance on MYC expression for BTZ sensitivity and that suppressed MYC expression is associated with a reduction in BTZ sensitivity, we found that pharmacologically suppressed MYC expression with CPI203 resulted in enhanced sensitivity to BTZ. Further, unlike a recently described report [8], we did not find that the BTZ resistant phenotype was associated with MYC pathway upregulation, as the basal MYC expression in the BTZ resistant cell line was actually lower than its wild type parent cell line.

The synergistic mechanisms of the BTZ combinations that have been studied also seem to vary. The effect of combining a deacetylase inhibitor (ie panobinostat or vorinostat) with BTZ in MM has been described [19, 40, 41]. It has been shown that upon proteasome inhibition, MM cells utilize the aggresome pathway in an attempt to manage the excess misfolded proteins to avoid apoptosis. HDAC6 is involved in the acetylation of alpha-tubulin and facilitates shuttling of the aggresome to the lysosome for degradation. HDAC6 inhibitors interfere with aggresome function, leading to NOXA mediated apoptosis upon activation of cellular stress responses in part via caspase activity. The synergistic effects of BTZ + vorinostat was found to be related to increased NOXA induction and was additionally dependent on MYC expression [40], again unlike what we found for BTZ + CPI203. Further, reports describing the major transcriptional changes related to treatment with these agents appear to differ significantly [6, 7, 9, 42]. Lastly, the DNA methyltransferase inhibitor, 5-azacitidine has been found to synergize with BTZ, and similar to deacetylase inhibitors, the apoptotic effects of 5-azacitidine involves NOXA upregulation [43]. Others have reported BTZ mediated apoptosis can occur despite silencing of NOXA and can even occur in the MYC depleted setting, though the degree of apoptosis was reduced [30, 31, 40]. Other NOXA independent apoptotic pathways exist and are likely biologically relevant, though details of these are not well understood.

Our data suggests that the previously reported reliance on MYC expression for NOXA-mediated apoptosis is more complicated, and likely involves other mediators. Accumulating data on role of MYC in BTZ resistance supports the notion that there is an intricate balance of MYC expression levels, and the influence of other mediators may tip the balance toward either a proliferative or apoptotic phenotype. Based on our data, we may simply hypothesize that CPI203 downregulates MYC enough to preferentially reduce proliferation and aggresome mediated survival, yet permitting enough NOXA expression for BTZ potentiated apoptosis. However, given the far-reaching transcriptional effects of BET and the pleotropic effects of proteasome inhibition, the synergistic relationship observed is likely far more complex. While the precise mechanism of the synergistic relationship is not clearly understood at this time, the combination of BTZ + CPI203 shows compelling responses that are consistent across multiple *in vitro* settings. Collectively our findings provide support for the clinical investigation of combined BET and proteasome inhibition in drug resistant MM.

## MATERIALS AND METHODS

### Cells and cell culture

The characteristics and sources of the human MM cell lines used are depicted in Table 2. All cell lines were obtained from sources within 6 months of use. The BTZ and melphalan resistant cell lines (ANBL6 BR, 8226.BR, and 8226/LR5) were developed as previously described [33, 44]. Specifically, ANBL6 BR and 8226.BR were previously subjected to gene expression profiling by source authors and was found to have a number of genomic changes and enhanced susceptibility to IGF-1R blockade as compared to their wild type parent lines, ANBL6 WT and RPMI 8226 [33]. While gene expression profiling was not repeated, our confirmation of enhanced IGF-1R sensitivity provides evidence of authentication of these cell lines (see Results section above). All cell lines were grown in R10 media, consisting of RPMI-1640 medium supplemented with 10% FBS, 100 units/mL penicillin, and 100 μg/mL streptomycin (Life Technologies). Media was supplemented with 1ng/mL of human recombinant IL-6 (Peprotech) for IL-6 dependent cell lines (ANBL6 WT and BR). ANBL6 BR and 8226.BR were grown in the presence of 10 nM bortezomib (Selleck Chemicals) while 8226/LR5 was grown in the presence of 5 μM melphalan (Sigma).

**Table 2:** Human myeloma cell lines used with corresponding characteristics and sources

Cell Line	Source	Bortezomib Resistant	Melphalan Resistant	Wild Type Parent Line
**U266**	American Type Culture Collection	No	No	N/A
**RPMI 8226**	American Type Culture Collection, Dr. Robert Orlowski (MD Anderson Cancer Center, Houston, TX), and Dr. William Dalton (H. Lee Moffitt Cancer Center, Tampa, FL)	No	No	N/A
**ANBL6 WT**	Dr. Robert Orlowski (MD Anderson Cancer Center, Houston, TX)	No	No	N/A
**8226/LR5**	Dr. William Dalton (H. Lee Moffitt Cancer Center, Tampa, FL)	No	Yes	RPMI 8226
**8226.BR**	Dr. Robert Orlowski (MD Anderson Cancer Center, Houston, TX)	Yes	No	RPMI 8226
**ANBL6 BR**	Dr. Robert Orlowski (MD Anderson Cancer Center, Houston, TX)	Yes	No	ANBL6 WT

The BTZ and melphalan resistant cell lines (ANBL6 BR, 8226.BR, and 8226/LR5) were developed as previously described [33, 44]. Note that RPMI 8226 (wild type) was obtained from 3 sources, in each comparison between a drug resistant line and its wild type counterpart; we used the wild type RPMI 8226 cells obtained from the same source as the drug resistant cells to which it was compared, unless otherwise noted.

### Primary bone marrow sample preparation

Primary cells from a patient with relapsed-refractory MM was collected by bone marrow aspiration with informed consent of the patient under a protocol approved by the institutional review board at Oregon Health and Science University. The bone marrow aspirate underwent independent clinical pathologic review and was composed of 90% myeloma cells. Red cell lysis of the bone marrow sample with Ammonium-Chloride-Potassium (ACK) buffer was performed. Given the significant myeloma cell population, and to preserve the marrow microenvironment, CD138 selection of tumor cells was omitted. The primary bone marrow cells were seeded at a concentration of 3.0 × 10^5^ cells/mL and incubated for 48 hours in R10 media supplemented with 1ng/mL IL-6, then tested for cell viability using the CellTiter 96 Aqueous One Solution Cell proliferation assay (Promega).

### Cell line small-molecule inhibitor plates and cell viability assay

Cell lines were seeded in 96-well plates at a concentration of 3.0 × 10^4^ cells/mL in 50 μL of media per well and incubated for 72 hours. All cell lines were initially screened using a panel of small molecule inhibitors as previously described [3]. All drugs were obtained from commercial vendors with the exception of CPI203, which was generously provided by Constellation Pharmaceuticals. Supplementary Table S1 lists the small-molecule inhibitors included on the screening plate as well as their targets and the sources from which they were obtained. All drugs were dissolved and stored in DMSO. In all cell culture experiments the final concentration of DMSO used was ≤0.1%. Unless otherwise noted, when testing two drugs for synergy, the two drug combinations were plated in serial constant ratio concentrations in 96-well plates with a HP D300 Digital Dispenser. Cell viability testing was performed on the small-molecule inhibitor screening plates and synergy plates using the CellTiter 96 Aqueous One Solution Cell proliferation assay (Promega) as previously described [3].

### Apoptosis assays

Cells were stained for Annexin V-PE/7-AAD as per manufacturer instructions (Guava Nexin, Millipore) after mechanical resuspension. The relative percentage of cells undergoing apoptosis was then quantified by flow cytometric analysis (Guava PCA flow cytometer, Millipore). All apoptotic measurements were performed in duplicate.

### Immunoblotting

Cell lysis buffer (Cell Signaling) with phenylmethanesulfonyl fluoride (PMSF) (Sigma), aprotinin (Sigma), and protease inhibitor cocktail (Sigma) was used to make cell lysates. Proteins were separated by electrophoresis on Tris-HCL precast gels (Bio-Rad Laboratories). Proteins were transferred to PVDF membranes (Immobilon-P, Millipore) with pore size 0.45um for MYC and 0.22um for NOXA blots then probed with antibodies against MYC (Cell Signaling), NOXA (Calbiochem), actin (Millipore), and GAPDH (Ambion). Chemiluminescence detection was performed using Clarity Western ECL substrate (Bio-Rad) or SuperSignal West Femto (Thermo Scientific) and viewed using ChemiDoc MP imaging system with Image Lab software (Bio-Rad Laboratories, Version 4.1).

### Statistical analyses

Data presented as means of duplicate or triplicate samples or assays. The student t-test was used to determine statistical difference between the groups. Combination index (CI) analyses were performed using Calcusyn software (Version 2.1, Biosoft). A CI value of 1 indicates additive effect, a CI of >1 indicates antagonistic effect, and a CI of <1 indicates synergy. Based on synergy growth curves, calculated CI values are reported at the effective doses of the combination resulting in 50%, 75%, and 90% inhibition (ED50, ED75, and ED90 respectively) [45].

## SUPPLEMENTARY FIGURES AND TABLES


