# Supplementary material for: Small molecule inhibitor screen identifies synergistic activity of the bromodomain inhibitor CPI203 and bortezomib in drug resistant myeloma
Source: Oncotarget. 2015 May 20;6(22):18921–32. doi: 10.18632/oncotarget.4214 (PMC4662464; doi:10.18632/oncotarget.4214)
Supplement: Supplementary file 1 [file oncotarget-06-18921-s001.pdf]

Small molecule inhibitor screen identifies synergistic activity of the bromodomain inhibitor CPI203 and bortezomib in drug resistant myeloma

Supplementary Material

Supplementary Table S1: Small molecule inhibitors. Listing of all 116 agents on the screening inhibitor panel with respective molecular targets and sources.

Drug name	Target	Source
17-AAG	Hsp90	LC Labs, PKC Pharmaceuticals
A-674563	AKT	Selleck
AB-1010= masitinib	KIT/PDGFR	LC Labs, PKC Pharmaceuticals
ABT-737	Bcl-2	Selleck
ABT-869= linifanib	VEGFR/PDGFR/KDR/CSF1R	Selleck
AC-220_2= quizartinib	FLT3	LC Labs, PKC Pharmaceuticals
AC-220_3= quizartinib	FLT3	LC Labs, PKC Pharmaceuticals
AC-220= quizartinib	FLT3	LC Labs, PKC Pharmaceuticals
AG-013736= axitinib	VEGFR1,2,3/PDGFR/cKIT	LC Labs, PKC Pharmaceuticals
AKT IV	AKT	EMD Chemicals
AMG-706= motesanib	VEGFR1,2,3/PDGFR/cKIT/RET	LC Labs, PKC Pharmaceuticals
AMPK	AMPK/KDR/VEGFR2/ALK2/BMPR1	EMD Chemicals
AP24534_2= ponatinib	BCR/ABL T315I /FLT3/RET/KIT/FGFR/PDGFR	LC Labs, PKC Pharmaceuticals
AP24534_3= ponatinib	BCR/ABL T315I /FLT3/RET/KIT/FGFR/PDGFR	LC Labs, PKC Pharmaceuticals
AP24534= ponatinib	BCR/ABL T315I /FLT3/RET/KIT/FGFR/PDGFR	LC Labs, PKC Pharmaceuticals
AT-7519	CDK	Selleck
AV-951= tivozanib	VEGFR	Selleck
AZD0530= saracatinib	Src/Fyn/Lyn/Blk/Fgr/Lck	Selleck
AZD-1152= barasertib	Aurora B	Selleck
AZD-1480	JAK-2	Selleck
AZD-2171= cediranib	VEGFR	Selleck
AZD-6244= selumetinib	MEK1	LC Labs, PKC Pharmaceuticals
BAY73-4506= regorafenib	c-KIT/VEGFR1,2/B-Raf/RET/PDGFR	Selleck
BEZ235	PI3K/mTOR	LC Labs, PKC Pharmaceuticals
BI-2536	PLK1	Selleck
BIBW-2992= afatinib	ERB2/EGFR	Selleck
BIRB-796= doramapimod	MAPK	LC Labs, PKC Pharmaceuticals
BMS-345541	IKK	Selleck
BMS-387032	CDK2	Selleck
Bortezomib	proteasome inhibitor	LC Labs, PKC Pharmaceuticals
Cal101	PI3K	Selleck
CEP-701= lestaurtinib	FLT3/JAK2/Trk	LC Labs, PKC Pharmaceuticals
CHIR-258= dovitinib	FLT3/c-KIT/FGFR1,3/VEGFR1,2,3/PDGFR/CSF1R	LC Labs, PKC Pharmaceuticals
CHIR-265	B-RAF/VEGFR	Selleck
CHIR-99021	GSK3-B	LC Labs, PKC Pharmaceuticals

CI-1033= canertinib	EGFR/ERB2	LC Labs, PKC Pharmaceuticals
CP-690550= tofacitinib	JAK	LC Labs, PKC Pharmaceuticals
CPI-267203	BET	Constellation Pharmaceuticals
Crenolanib	PDGFRA/B	Selleck
Crenolanib_2	PDGFRA/B	Selleck
Crenolanib_3	PDGFRA/B	Selleck
CYC-202	cdc/cdk/cyclin	LC Labs, PKC Pharmaceuticals
Cytopia= CYT387	JAK1/JAK2	Selleck
Dasatinib	BCR/ABL, SRC, c-Kit	LC Labs, PKC Pharmaceuticals
Dasatinib_2	BCR/ABL, SRC, c-Kit	LC Labs, PKC Pharmaceuticals
Dasatinib_3	BCR/ABL, SRC, c-Kit	LC Labs, PKC Pharmaceuticals
EKB-569= pelitinib	ERB1,2,4	Selleck
Elesclomol	Hsp90/apoptosis	Selleck
Erlotinib	EGFR/JAK2V617F	LC Labs, PKC Pharmaceuticals
Erlotinib_2	EGFR/JAK2V617F	LC Labs, PKC Pharmaceuticals
Flavopiridol= alvocidib	cdk	Selleck
GDC-0449= vismodegib	hedgehog	LC Labs, PKC Pharmaceuticals
GDC-0879	B-RAFV600E/pERK	Selleck
GDC-0941	PI3K	LC Labs, PKC Pharmaceuticals
GDC-0941_2	PI3K	LC Labs, PKC Pharmaceuticals
Gefitinib	EGFR	LC Labs, PKC Pharmaceuticals
Gefitinib_2	EGFR	LC Labs, PKC Pharmaceuticals
Go6976	PKC	LC Labs, PKC Pharmaceuticals
GSK-1120212	MEK1/2	Selleck
GSK-1838705A	IGF1R and ALK	Selleck
GSK-1904529A	IGF1R and IR	Selleck
GSK-1904529A_2	IGF1R and IR	Selleck
GSK-690693	AKT	Selleck
GW-2580	CSF1R	LC Labs, PKC Pharmaceuticals
GW-2580_2	CSF1R	LC Labs, PKC Pharmaceuticals
GW-786034_2= pazopanib	VEGFR/c-KIT	Selleck
GW-786034= pazopanib	VEGFR/c-KIT	Selleck
H-89	PKA	LC Labs, PKC Pharmaceuticals
HKI-272= neratinib	ERB2/EGFR	Selleck
Imatinib	BCR/ABL, KIT	Selleck
INCB018424_2= ruxolitinib	JAK	LC Labs, PKC Pharmaceuticals
INCB018424= ruxolitinib	JAK	LC Labs, PKC Pharmaceuticals
INK-128	mTOR	Selleck
JNJ-28312141	CSF1R/FLT3	SYN Kinase
JNJ-38877605	c-MET	Selleck
JNJ-7706621	CDK/cyclin, AuroraA and B	Selleck
JNK II	c-JUN	LC Labs, PKC Pharmaceuticals
KI-20227	CSF1R, VEGFR, c-KIT PDGFRB	Tocris (R&D)
KN92	CAMKII	VWR (Calbiochem)

KN93	CAMKII	EMD Chemicals
KU-55933	PI3K/mTOR/ATM	Selleck
KW-2449	FLT3/ABL/FGFR1/AuroraA	Selleck
Lapatinib	ERB2/EGFR	Selleck
Lapatinib_2	ERB2/EGFR	Selleck
LY294002	PI3K	Selleck
LY-333531= ruboxistaurin	PKCB	Tocris
MGCD-265	MET/VEGFR/Tie2	Selleck
MK-2206	AKT1,2,3	Selleck
MLN-120B	IKKB	MedChem Exp
MLN-518= tandutinib	FLT3, PDGFRB, KIT	LC Labs, PKC Pharmaceuticals
MLN-8054	Aurora A	Selleck
MLN-8237= alisertib	Aurora A	Selleck
NF-kB	NF-kB	EMD Chemicals
Nilotinib	BCR-ABL/KIT/LCK/EPHA/DDR	Selleck
Nilotinib_2	BCR-ABL/KIT/LCK/EPHA/DDR	Selleck
Nilotinib_3	BCR-ABL/KIT/LCK/EPHA/DDR	Selleck
NVP-ADW742	IGF1R	Selleck
NVP-ADW742_2	IGF1R	Selleck
NVP-TAE-684	ALK	Selleck
p38	p38	VWR (Calbiochem)
pan-JAK	JAK	EMD Chemicals
PD-173955	BCR-ABL, SRC	Symansis
PD-184352	MEK/MAPK	LC Labs, PKC Pharmaceuticals
PF-2341066_2= crizotinib	ALK	LC Labs, PKC Pharmaceuticals
PF-2341066= Crizotinib	ALK	LC Labs, PKC Pharmaceuticals
PHA-665752	c-Met	Selleck
PHT-427	AKT, PDPK1	Selleck
PI-103	P13K	Selleck
PKC-412= midostaurin	PKC	LC Labs, PKC Pharmaceuticals
PLX-4720	Raf, BRAFV600E, CRAFY340D/Y341D	Selleck
PLX-4720_2	Raf, BRAFV600E, CRAFY340D/Y341D	Selleck
PP2	Src family, LCK, FYN, HCK	VWR (Calbiochem)
PP242	mTOR	LC Labs, PKC Pharmaceuticals
PRT062607	Syk	Selleck
PTK-787= vatalanib	VEGFR, KIT, PDGFR	LC Labs, PKC Pharmaceuticals
Rapamycin_2= sirolimus	mTOR, IL2	LC Labs, PKC Pharmaceuticals
Rapamycin= sirolimus	mTOR, IL2	LC Labs, PKC Pharmaceuticals
S31-201= NSC 74859	STAT3	Santa Cruz Biotech
SB-202190	MAPK	LC Labs, PKC Pharmaceuticals
SB-203580	MAPK	LC Labs, PKC Pharmaceuticals
SB-431542	ALK5	Selleck
SGX-523	MET	Selleck
SKI-606= bosutinib	Src, Abl	LC Labs, PKC Pharmaceuticals

Sorafenib	VEGFR, PDGFR, RAF	LC Labs, PKC Pharmaceuticals
Sorafenib_2	VEGFR, PDGFR, RAF	LC Labs, PKC Pharmaceuticals
Sorafenib_3	VEGFR, PDGFR, RAF	LC Labs, PKC Pharmaceuticals
Staurosporin	PKC, wide range of targets	LC Labs, PKC Pharmaceuticals
STO609	CAMKK	EMD Millipore
SU11274	MET	Selleck
Sunitinib	PDGFR, VEGFR, KIT, RET, CSF1R, FLT3	LC Labs, PKC Pharmaceuticals
Sunitinib_2	PDGFR, VEGFR, KIT, RET, CSF1R, FLT3	LC Labs, PKC Pharmaceuticals
Sunitinib_3	PDGFR, VEGFR, KIT, RET, CSF1R, FLT3	LC Labs, PKC Pharmaceuticals
TG-100-115	PI3K	Selleck
TG-101348	JAK2, JAK2V617F, FLT3, RET	Active Biochem
Vargetef= nintendanib	VEGFR, PDGFR, FGFR	LC Labs, PKC Pharmaceuticals
VX-680= Tozasertib	pan-Aurora	LC Labs, PKC Pharmaceuticals
VX-745	p-38a MAPK	Tocris
XAV-939	TNKS1, 2 (wnt b-catenin pathway)	Selleck
XL184= cabozanitinib	MET and VEGFR2	Selleck
XL-880	MET, VEGFR2,KDR	LC Labs, PKC Pharmaceuticals
YM-155	survivin	Selleck
ZD-6474= Vandetanib	VEGFR, EGFR, RET	LC Labs, PKC Pharmaceuticals

Supplementary Table S2: Complete listing of R50 values- with available corresponding median patient IC50 values, and median MM cell line IC50 values. R50 values represent the pooled patient sample median IC50 value divided by the median IC50 value from the myeloma cell lines tested. Therefore, the R50 value represents the fold magnitude increased sensitivity to a drug in the MM cell lines compared to the pooled database control. IC50 data for each drug for each patient specimen used to generate the median patient IC50 values have been previously published [3]. Note that the RPMI 8226 IC50 values used represents a mean the wild type RPMI 8226 strains that were obtained from 3 separate sources as described in Table 2. IC50 values are listed as nanomolar units.

R50 (median pt IC50 ÷ median MM cell line IC50)	Median patient IC50 from ref (3) (nM)	Median IC50 of pooled MM lines (nM)	RPMI 8226 IC50 (nM)	8226.BR IC50 (nM)	8226/LR5 IC50 (nM)	ANBL6 WT IC50 (nM)	ANBL6 BR IC50 (nM)	U266 IC50 (nM)	Drug name	Target
17.1	6888.0	404.0	371.7	417.9	390.0	661.7	282.8	10000.0	PKC-412= midostaurin	PKC
15.5	108.0	7.0	6.2	7.5	6.0	7.9	6.5	27.3	Staurosporin	PKC, wide range of targets
14.1	10000.0	710.6	903.2	913.0	759.6	209.9	661.7	617.6	MLN-8054	Aurora A
12.1	10000.0	824.9	418.5	1231.3	159.5	129.8	10000.0	10000.0	VX-680= Tozasertib	pan-Aurora
6.0	2217.0	368.5	167.4	96.5	677.1	617.6	289.4	447.7	PI-103	PI3K
4.3	1475.0	340.2	323.9	414.8	277.0	356.5	199.6	2834.9	AKT IV	AKT
2.3	10000.0	4257.6	6815.9	10000.0	1699.2	187.2	163.2	10000.0	Go6976	PKC
1.9	4307.0	2323.7	1434.5	1334.0	2229.5	9866.6	2417.9	6576.3	LY294002	PI3K
1.5	190.0	127.7	134.1	121.2	108.2	159.5	67.1	187.2	BMS-387032	CDK2
1.1	10000.0	8821.6	8670.1	5809.5	6156.1	8973.1	9637.8	10000.0	Sorafenib	VEGFR, PDGFR, RAF
1.1	10000.0	9107.4	8758.0	4802.9	9456.8	8083.5	10000.0	10000.0	GW-786034= pazopanib	VEGFR/c-KIT
1.1	10000.0	9149.8	8299.6	10000.0	10000.0	692.8	270.2	10000.0	NF-kB	NF-kB
1.0	10000.0	10000.0	10000.0	10000.0	10000.0	10000.0	10000.0	10000.0	AMG-706= motesanib	VEGFR1,2,3/PDGFR/cKIT/RET
1.0	10000.0	10000.0	3731.7	10000.0	110.6	10000.0	10000.0	10000.0	AZD-1152= barasertib	Aurora B
1.0	10000.0	10000.0	10000.0	10000.0	9121.1	10000.0	10000.0	10000.0	BIRB-796= doramapimod	MAPK
1.0	10000.0	10000.0	10000.0	10000.0	10000.0	10000.0	10000.0	10000.0	Cal101	PI3K
1.0	10000.0	10000.0	10000.0	10000.0	10000.0	10000.0	1480.1	10000.0	CP-690550= tofacitinib	JAK
1.0	10000.0	10000.0	10000.0	10000.0	10000.0	10000.0	10000.0	10000.0	CYC-202	cdc/cdk/cyclin
1.0	10000.0	10000.0	9080.9	10000.0	7037.0	10000.0	10000.0	10000.0	Erlotinib	EGFR/JAK2V617F
1.0	10000.0	10000.0	10000.0	10000.0	10000.0	10000.0	10000.0	10000.0	Gefitinib	EGFR
1.0	10000.0	10000.0	10000.0	10000.0	10000.0	10000.0	10000.0	10000.0	GW-2580	CSF1R
1.0	10000.0	10000.0	10000.0	2571.4	7763.5	10000.0	10000.0	10000.0	H-89	PKA
1.0	10000.0	10000.0	10000.0	10000.0	10000.0	10000.0	10000.0	10000.0	Imatinib	BCR/ABL, KIT
1.0	10000.0	10000.0	10000.0	10000.0	4787.3	10000.0	10000.0	10000.0	KN92	CAMKII
1.0	10000.0	10000.0	10000.0	10000.0	8318.6	10000.0	10000.0	10000.0	KN93	CAMKII
1.0	10000.0	10000.0	10000.0	10000.0	10000.0	10000.0	10000.0	10000.0	MLN-518= tandutinib	FLT3, PDGFRB, KIT
1.0	10000.0	10000.0	10000.0	10000.0	10000.0	10000.0	10000.0	10000.0	p38	p38
1.0	10000.0	10000.0	9924.5	10000.0	7944.3	10000.0	10000.0	10000.0	PTK-787= vatalanib	VEGFR, KIT, PDGFR
1.0	10000.0	10000.0	10000.0	10000.0	10000.0	10000.0	10000.0	10000.0	SB-202190	MAPK
1.0	10000.0	10000.0	10000.0	10000.0	10000.0	10000.0	10000.0	10000.0	SB-203580	MAPK
1.0	10000.0	10000.0	10000.0	10000.0	10000.0	10000.0	10000.0	10000.0	SB-431542	ALK5
1.0	10000.0	10000.0	10000.0	10000.0	10000.0	10000.0	10000.0	10000.0	STO609	CAMKK
1.0	1000.0	1000.0	1000.0	1000.0	1000.0	1000.0	1000.0	1000.0	Sunitinib	PDGFR, VEGFR, KIT, RET, CSF1R, FLT3
1.0	10000.0	10000.0	10000.0	10000.0	10000.0	10000.0	10000.0	10000.0	VX-745	p-38a MAPK
0.8	8159.0	10000.0	10000.0	9550.9	10000.0	10000.0	9550.9	10000.0	PP2	Src family, LCK, FYN, HCK
0.8	9814.0	12666.1	11682.5	13649.8	8618.6	8164.8	32435.8	44873.5	JNK II	c-JUN
0.7	2721.0	3697.8	3763.9	3802.9	3549.1	3631.8	1699.2	10000.0	Cytopia= CYT387	JAK1/JAK2
0.7	2193.0	3114.6	5074.7	10000.0	813.8	3716.4	1585.9	2512.9	EKB-569= pelitinib	ERB1,2,4
0.7	3427.0	5061.3	5444.3	6457.5	4678.4	3716.4	2885.0	7414.1	JNJ-7706621	CDK/cyclin, Aurora A&B
0.7	6476.0	9707.0	9853.5	9560.6	4757.9	8622.7	10000.0	10000.0	Lapatinib	ERB2/EGFR
0.6	6338.0	10000.0	10000.0	5129.6	10000.0	10000.0	10000.0	10000.0	ZD-6474= Vandetanib	VEGFR, EGFR, RET
0.6	2531.0	4135.4	3314.1	3236.9	3802.9	6607.9	4467.8	4467.8	LY-333531= ruboxistaurin	PKCB
0.6	6094	10000.0	7302.2	10000.0	10000.0	6310.6	10000.0	10000.0	CHIR-265	B-RAF/VEGFR
0.5	350.0	650.8	615.0	349.4	539.8	742.5	686.6	720.6	AP24534= ponatinib	BCR/ABL/FLT3/RET/KIT/FGFR

										/PDGFR
0.5	3333.0	6983.6	7656.6	5624.4	8318.6	6310.6	4898.8	10000.0	ABT-869= linifanib	VEGFR/PDGFR/KDR/CSF1R
0.4	3732.0	8346.3	10000.0	4169.7	5249.1	6919.3	9773.4	10000.0	CI-1033= canertinib	EGFR/ERB2
0.4	2582	6116.2	3981.7	5624.4	2952.2	10000.0	6607.9	6761.8	CHIR-258= dovitinib	FLT3/c-KIT/FGFR1/FGFR3/ VEGFR1-3/PDGFR/CSF1R
0.4	4022.0	10000.0	10000.0	10000.0	10000.0	10000.0	10000.0	10000.0	AMPK	AMPK/KDR/VEGFR2/ALK2/B MPR1
0.4	401.0	1000.0	1000.0	1000.0	1000.0	1000.0	1000.0	1000.0	Dasatinib	BCR/ABL, SRC, c-Kit
0.4	3611.0	10000.0	9834.7	10000.0	10000.0	10000.0	10000.0	10000.0	Nilotinib	BCR-ABL/KIT/LCK/EPHA/DDR
0.3	61.0	184.2	158.4	209.9	224.9	139.0	59.9	408.4	Flavopiridol= alvocidib	cdk
N/A	N/A	242.9	260.9	113.2	126.9	224.9	502.2	1738.8	17-AAG	Hsp90
N/A	N/A	2372.5	2513.1	1699.2	1549.8	2399.8	2345.2	3236.9	A-674563	AKT
N/A	N/A	10000.0	10000.0	10000.0	6026.6	10000.0	10000.0	10000.0	AB-1010= masitinib	KIT/PDGFR
N/A	N/A	5589.1	4720.6	6457.5	1585.9	10000.0	4678.4	10000.0	ABT-737	Bcl-2
N/A	N/A	6998.7	1884.2	3997.4	624.0	10000.0	10000.0	10000.0	AC-220= quizartinib	FLT3
N/A	N/A	2856.6	3200.3	1319.3	2512.9	1996.3	10000.0	10000.0	AG-013736= axitinib	VEGFR1,2,3/PDGFR/cKIT
N/A	N/A	199.9	189.9	224.9	209.9	252.2	126.9	105.7	AT-7519	CDK
N/A	N/A	5412.8	5926.7	1622.8	4898.8	4074.8	10000.0	10000.0	AV-951= tivozanib	VEGFR
N/A	N/A	10000.0	10000.0	9121.1	10000.0	10000.0	8512.4	10000.0	AZD-1480	JAK-2
N/A	N/A	5622.4	4281.1	2455.7	4787.3	10000.0	10000.0	6457.5	AZD-2171= cediranib	VEGFR
N/A	N/A	10000.0	5842.7	10000.0	10000.0	10000.0	10000.0	7763.5	AZD-6244= selumetinib	MEK1
N/A	N/A	10000.0	10000.0	10000.0	9550.9	10000.0	10000.0	6607.9	AZD0530= saracatinib	Src/Fyn/Lyn/Blk/Fgr/Lck
N/A	N/A	5651.6	5136.2	2755.2	3549.1	9121.1	6167.0	10000.0	BAY73-4506= regorafenib	c-KIT/VEGFR1,2/B-Raf/RET/PDGFR
N/A	N/A	52.4	41.0	38.2	92.2	82.3	48.9	56.0	BEZ235	PI3K/mTOR
N/A	N/A	7.9	11.1	11.7	4.8	9.7	4.6	6.1	BI-2536	PLK1
N/A	N/A	1938.9	2425.2	1231.3	1381.4	1585.9	2291.9	5129.6	BIBW-2992= afatinib	ERB2/EGFR
N/A	N/A	3662.1	3342.2	3982.1	4266.8	4571.9	3091.3	2755.2	BMS-345541	IKK
N/A	N/A	431.8	89.3	1446.4	4.2	170.8	692.8	759.6	Bortezomib	proteasome
N/A	N/A	88.7	172.8	116.5	140.6	60.9	23.0	56.0	CEP-701= lestaurtinib	FLT3/JAK2/Trk
N/A	N/A	10000.0	10000.0	10000.0	10000.0	10000.0	10000.0	10000.0	CHIR-99021	GSK3-B
N/A	N/A	394.6	479.1	142.3	196.0	6919.3	310.0	646.7	CPI203	BET
N/A	N/A	2658.5	2649.0	2668.0	1648.7	6472.2	2425.4	2957.9	Crenolanib	PDGFRA/B
N/A	N/A	19.8	16.7	22.9	7.2	48.9	7.6	53.5	Elesclomol	Hsp90/apoptosis
N/A	N/A	10000.0	10000.0	10000.0	10000.0	10000.0	10000.0	10000.0	GDC-0449= vismodegib	hedgehog
N/A	N/A	10000.0	10000.0	10000.0	10000.0	10000.0	10000.0	10000.0	GDC-0879	B-RAFV600E/pERK
N/A	N/A	1897.5	1141.7	267.5	7269.5	2395.3	1399.7	6149.5	GDC-0941	PI3K
N/A	N/A	10000.0	10000.0	10000.0	10000.0	10000.0	1024.3	15.1	GSK-1120212	MEK1/2
N/A	N/A	2692.5	2173.2	1319.3	2692.5	3468.4	2692.5	9773.4	GSK-1838705A	IGF1R and ALK
N/A	N/A	10000.0	9337.8	10000.0	10000.0	10000.0	10000.0	10000.0	GSK-1904529A	IGF1R and IR
N/A	N/A	10000.0	10000.0	10000.0	10000.0	10000.0	10000.0	10000.0	GSK-690693	AKT
N/A	N/A	872.1	2297.4	3802.9	390.0	1097.5	646.7	364.1	HKI-272= neratinib	ERB2/EGFR
N/A	N/A	10000.0	10000.0	10000.0	10000.0	10000.0	1286.8	10000.0	INCB018424= ruxolitinib	JAK
N/A	N/A	11.2	5.5	5.2	9.8	36.0	12.6	12.8	INK-128	mTOR
N/A	N/A	10000.0	10000.0	10000.0	10000.0	10000.0	10000.0	10000.0	JNJ-28312141	CSF1R/FLT3
N/A	N/A	10000.0	9850.3	10000.0	10000.0	10000.0	10000.0	10000.0	JNJ-38877605	c-MET
N/A	N/A	9666.8	10000.0	5755.4	8318.6	9333.5	10000.0	10000.0	KI-20227	CSF1R, VEGFR, c-KIT PDGFRB
N/A	N/A	10000.0	10000.0	10000.0	10000.0	10000.0	10000.0	10000.0	KU-55933	PI3K/mTOR/ATM
N/A	N/A	3759.6	3027.5	2885.0	3802.9	3716.4	4787.3	5129.6	KW-2449	FLT3/ABL/FGFR1/AurKA
N/A	N/A	9818.9	9637.8	6457.5	10000.0	10000.0	9333.5	10000.0	MGCD-265	MET/VEGFR/Tie2
N/A	N/A	8254.4	6508.8	2239.7	10000.0	4266.8	10000.0	10000.0	MK-2206	AKT1,2,3
N/A	N/A	5000.0	5000.0	5000.0	5000.0	5000.0	5000.0	5000.0	MLN-120B	IKKB
N/A	N/A	108.8	133.5	502.2	48.9	14.5	84.2	1097.5	MLN-8237= alisertib	Aurora A
N/A	N/A	5352.3	6763.9	2887.6	10000.0	5204.3	5500.2	4240.6	NVP-ADW742	IGF1R
N/A	N/A	480.1	508.0	214.8	296.1	502.2	458.1	603.6	NVP-TAE-684	ALK
N/A	N/A	7547.5	7849.7	7245.4	10000.0	4074.8	364.1	10000.0	pan-JAK	JAK
N/A	N/A	2353.9	3122.0	1514.6	1319.3	4678.4	1585.9	4366.2	PD-173955	BCR-ABL, SRC
N/A	N/A	6358.2	5021.2	7586.8	4898.8	8129.3	10000.0	5129.6	PD-184352	MEK/MAPK
N/A	N/A	5309.2	6756.0	4240.6	6106.5	6072.1	3163.3	4546.4	PF-2341066= Crizotinib	ALK
N/A	N/A	4845.6	5167.0	4678.4	3982.1	10000.0	5012.9	3236.9	PHA-665752	c-Met
N/A	N/A	10000.0	10000.0	10000.0	10000.0	10000.0	10000.0	10000.0	PHT-427	AKT, PDPK1
N/A	N/A	9887.7	10000.0	10000.0	9256.2	10000.0	9775.5	7944.7	PLX-4720	Raf, BRAFV600E, CRAF
N/A	N/A	243.7	204.7	148.9	417.9	617.6	246.5	240.9	PP242	mTOR
N/A	N/A	3394.1	3398.7	2455.7	3631.8	3021.0	10000.0	3389.4	PRT062607	Syk
N/A	N/A	2718.8	173.6	129.2	432.2	5035.1	5005.4	5062.0	Rapamycin= sirolimus	mTOR, IL2

N/A	N/A	10000.0	10000.0	10000.0	10000.0	10000.0	10000.0	10000.0	S31-201= NSC 74859	STAT3
N/A	N/A	10000.0	9924.5	10000.0	10000.0	10000.0	10000.0	10000.0	SGX-523	MET
N/A	N/A	6543.1	4284.6	6167.0	2090.3	10000.0	6919.3	9121.1	SKI-606= bosutinib	Src, Abl
N/A	N/A	9456.8	5920.7	10000.0	8913.5	10000.0	4678.4	10000.0	SU11274	MET
N/A	N/A	10000.0	10000.0	10000.0	10000.0	10000.0	10000.0	10000.0	TG-100-115	PI3K
		1565.1	1469.5	1123.0	381.2	6607.9	1660.6	1779.3	TG-101348	JAK2, JAK2V617F, FLT3, RET
N/A	N/A	7162.9	8262.4	6026.6	10000.0	7245.4	7080.5	1350.0	Vargetef= nintendanib	VEGFR, PDGFR, FGFR
N/A	N/A	10000.0	10000.0	10000.0	10000.0	10000.0	10000.0	10000.0	XAV-939	TNKS1, 2 (wnt b-catenin pathway)
N/A	N/A	1047.8	1380.9	576.4	777.2	1203.3	892.3	1863.1	XL-880	MET, VEGFR2,KDR
N/A	N/A	8381.5	6763.0	3982.1	6026.6	10000.0	10000.0	10000.0	XL184= cabozanitinib	MET and VEGFR2
N/A	N/A	6.2	3.5	8.9	2.8	13.9	4.4	7.9	YM-155	survivin

Supplementary Table S3: Complete listing of paired melphalan- resistant and –sensitive IC50 values. The values listed under WT IC50 ÷ LR5 IC50 represents the fold magnitude increased sensitivity in melphalan resistant cell lines as compared to their wild type parent cell lines (IC50s for RPMI 8226 WT and 8226/LR5. These values were obtained by dividing the IC50 values for the wild type RPMI 8226 WT by the corresponding IC50 value of the melphalan resistant cell line, 8226/LR5. The table includes in rank order for all 116 drugs with the greatest increase in sensitivity in the BR cell lines.

RPMI 8226 IC50 ÷ 8226/LR5 IC50	RPMI 8226 IC50 (nM)	8226/LR5 IC50 (nM)	**Drug name**	**Target**
90.4	10000.0	110.6	AZD-1152= barasertib	Aurora B
12.3	51.1	4.2	Bortezomib	proteasome
6.7	2631.3	390.0	HKI-272= neratinib	ERB2/EGFR
5.9	10000.0	1699.2	Go6976	PKC
5.6	4571.9	813.8	EKB-569= pelitinib	ERB1,2,4
5.5	8710.6	1585.9	ABT-737	Bcl-2
3.9	1480.1	381.2	TG-101348	JAK2, JAK2V617F, FLT3, RET
3.9	490.8	126.9	17-AAG	Hsp90
3.1	490.8	159.5	VX-680= Tozasertib	pan-Aurora
2.7	129.8	48.9	MLN-8237= alisertib	Aurora A
2.6	7763.5	2952.2	CHIR-258= dovitinib	FLT3/cKIT/FGFR1,3/VEGFR1,2,3,/PDGFR/CSF1R
2.4	17.2	7.2	Elesclomol	Hsp90/apoptosis
2.4	20479.8	8618.6	JNK II	c-JUN
2.3	458.1	196.0	CPI203	BET
2.1	10000.0	4757.9	Lapatinib	ERB2/EGFR
2.1	10000.0	4787.3	KN92	CAMKII
2.0	10000.0	4898.8	AV-951= tivozanib	VEGFR
1.9	1216.3	624.0	AC-220= quizartinib	FLT3
1.9	9.3	4.8	BI-2536	PLK1
1.9	10000.0	5249.1	CI-1033= canertinib	EGFR/ERB2
1.9	2631.3	1381.4	BIBW-2992= afatinib	ERB2/EGFR
1.8	2399.8	1319.3	PD-173955	BCR-ABL, SRC
1.7	3549.1	2090.3	SKI-606= bosutinib	Src, Abl
1.7	183.0	108.2	BMS-387032	CDK2
1.7	10000.0	6026.6	AB-1010= masitinib	KIT/PDGFR
1.6	1259.9	777.2	XL-880	MET, VEGFR2,KDR
1.6	2624.8	1648.7	Crenolanib	PDGFRA/B
1.6	1203.3	759.6	MLN-8054	Aurora A
1.5	4.2	2.8	YM-155	survivin
1.4	10000.0	7037.0	Erlotinib	EGFR/JAK2V617F
1.4	8304.0	6106.5	PF-2341066= Crizotinib	ALK
1.3	10000.0	7763.5	H-89	PKA
1.2	9773.4	7944.3	PTK-787= vatalanib	VEGFR, KIT, PDGFR
1.2	5755.4	4678.4	JNJ-7706621	CDK/cyclin, Aurora A&B
1.2	7458.3	6156.1	Sorafenib	VEGFR, PDGFR, RAF
1.2	1863.1	1549.8	A-674563	AKT
1.2	10000.0	8318.6	KI-20227	CSF1R, VEGFR, c-KIT PDGFRB
1.2	10000.0	8318.6	KN93	CAMKII
1.1	4074.8	3549.1	BAY73-4506= regorafenib	c-KIT/VEGFR1,2/B-Raf/RET/PDGFR
1.1	6761.8	6026.6	XL184= cabozanitinib	MET and VEGFR2
1.1	10000.0	9121.1	BIRB-796=	MAPK

			doramapimod	
1.1	10000.0	9256.2	PLX-4720	Raf, BRAFV600E, CRAF
1.1	3802.9	3549.1	Cytopia= CYT387?	JAK1/JAK2
1.0	10000.0	9550.9	AZD0530= saracatinib	Src/Fyn/Lyn/Blk/Fgr/Lck
1.0	548.4	539.8	AP24534= ponatinib	BCR/ABL/FLT3/RET/KIT/FGFR/PDGFR
1.0	6.0	6.0	Staurosporin	PKC, wide range of targets
1.0	5000.0	5000.0	MLN-120B	IKKB
1.0	10000.0	10000.0	Cal101	PI3K
1.0	10000.0	10000.0	GDC-0879	B-RAFV600E/pERK
1.0	10000.0	10000.0	p38	p38
1.0	10000.0	10000.0	SB-203580	MAPK
1.0	1000.0	1000.0	Sunitinib	PDGFR, VEGFR, KIT, RET, CSF1R, FLT3
1.0	10000.0	10000.0	MLN-518= tandutinib	FLT3, PDGFRB, KIT
1.0	10000.0	10000.0	TG-100-115	PI3K
1.0	10000.0	10000.0	GSK-690693	AKT
1.0	10000.0	10000.0	SB-202190	MAPK
1.0	10000.0	10000.0	GW-2580	CSF1R
1.0	10000.0	10000.0	STO609	CAMKK
1.0	10000.0	10000.0	PP2	Src family, LCK, FYN, HCK
1.0	10000.0	10000.0	AMG-706= motesanib	VEGFR1,2,3/PDGFR/cKIT/RET
1.0	10000.0	10000.0	VX-745	p-38a MAPK
1.0	10000.0	10000.0	CYC-202	cdc/cdk/cyclin
1.0	10000.0	10000.0	JNJ-28312141	CSF1R/FLT3
1.0	10000.0	10000.0	AMPK	AMPK/KDR/VEGFR2/ALK2/BMPR1
1.0	10000.0	10000.0	SB-431542	ALK5
1.0	10000.0	10000.0	CP-690550= tofacitinib	JAK
1.0	10000.0	10000.0	AZD-1480	JAK-2
1.0	10000.0	10000.0	Gefitinib	EGFR
1.0	10000.0	10000.0	Imatinib	BCR/ABL, KIT
1.0	1000.0	1000.0	Dasatinib	BCR/ABL, SRC, c-Kit
1.0	10000.0	10000.0	ZD-6474= Vandetanib	VEGFR, EGFR, RET
1.0	10000.0	10000.0	CHIR-99021	GSK3-B
1.0	10000.0	10000.0	GDC-0449= vismodegib	hedgehog
1.0	10000.0	10000.0	KU-55933	PI3K/mTOR/ATM
1.0	10000.0	10000.0	PHT-427	AKT, PDPK1
1.0	10000.0	10000.0	S31-201= NSC 74859	STAT3
1.0	10000.0	10000.0	XAV-939	TNKS1, 2 (wnt b-catenin pathway)
1.0	10000.0	10000.0	ruxolitinib	JAK
1.0	10000.0	10000.0	JNJ-38877605	c-MET
1.0	10000.0	10000.0	Nilotinib	BCR-ABL/KIT/LCK/EPHA/DDR
1.0	10000.0	10000.0	GSK-1904529A	IGF1R and IR
1.0	10000.0	10000.0	Vargetef= nintendanib	VEGFR, PDGFR, FGFR
1.0	10000.0	10000.0	pan-JAK	JAK
1.0	10000.0	10000.0	CHIR-265	B-RAF/VEGFR
1.0	10000.0	10000.0	NVP-ADW742	IGF1R
1.0	10000.0	10000.0	GSK-1120212	MEK1/2
1.0	9773.4	10000.0	SGX-523	MET
0.9	250.4	277.0	AKT IV	AKT
0.9	8913.5	10000.0	MGCD-265	MET/VEGFR/Tie2
0.9	3468.4	3982.1	PHA-665752	c-Met
0.9	8083.5	9456.8	GW-786034= pazopanib	VEGFR/c-KIT
0.8	174.8	209.9	AT-7519	CDK
0.8	3163.3	3802.9	LY-333531= ruboxistaurin	PKCB

0.8	3468.4	4266.8	BMS-345541	IKK
0.8	310.0	390.0	PKC-412= midostaurin	PKC
0.8	6761.8	8913.5	SU11274	MET
0.7	1820.7	2512.9	AG-013736= axitinib	VEGFR1,2,3/PDGFR/cKIT
0.7	2755.2	3802.9	KW-2449	FLT3/ABL/FGFR1/AurKA
0.7	7080.5	10000.0	AZD-6244= selumetinib	MEK1
0.7	5889.4	8318.6	ABT-869= linifanib	VEGFR/PDGFR/KDR/CSF1R
0.7	152.4	224.9	Flavopiridol= alvocidib	cdk
0.7	1486.3	2229.5	LY294002	PI3K
0.7	6607.9	10000.0	MK-2206	AKT1,2,3
0.6	2345.2	3631.8	PRT062607	Syk
0.6	2952.2	4787.3	AZD-2171= cediranib	VEGFR
0.6	3021.0	4898.8	PD-184352	MEK/MAPK
0.5	159.5	296.1	NVP-TAE-684	ALK
0.5	73.4	140.6	CEP-701= lestaurtinib	FLT3/JAK2/Trk
0.5	4898.8	10000.0	NF-kB	NF-kB
0.5	1289.2	2692.5	GSK-1838705A	IGF1R and ALK
0.5	4.4	9.8	INK-128	mTOR
0.4	34.1	92.2	BEZ235	PI3K/mTOR
0.3	142.3	417.9	PP242	mTOR
0.3	145.1	432.2	Rapamycin= sirolimus	mTOR, IL2
0.3	191.5	677.1	PI-103	PI3K
0.1	1072.8	7269.5	GDC-0941	PI3K

Supplementary Table S4: Complete listing of paired bortezomib- resistant and – sensitive IC50 values. The values listed under WT IC50 ÷ BR IC50 represents the fold magnitude increased sensitivity in the BTZ resistant cell lines as compared to their wild type parent cell lines (IC50s for ANBL6 WT ÷ ANBL6 BR, and RPMI 8226 ÷ 8226.BR). These values were obtained by dividing the IC50 values for the wild type MM cell lines (RPMI 8226 and ANBL6 WT) by the corresponding IC50 value of the BTZ resistant cell line.

WT IC50 ÷ BR IC50								
AVG (RPMI & ANBL6)	ANBL6	RPMI	RPMI 8226 IC50 (nM)	8226.BR IC50 (nM)	ANBL6 WT IC50 (nM)	ANBL6 BR IC50 (nM)	Drug name	Target
16.98	22.32	5.34	759.6	142.3	6919.3	310.0	CPI203	BET
3.02	4.08	1.08	1446.6	1334.0	9866.6	2417.9	LY294002	PI3K
3.01	3.98	1.58	1779.3	1123.0	6607.9	1660.6	TG-101348	JAK2, JAK2V617F, FLT3, RET
2.74	3.76	1.01	5.9	6.2	70.2	10.8	Rapamycin	mTOR, IL2
2.74	2.95	2.51	3802.9	1514.6	4678.4	1585.9	PD-173955	BCR-ABL, SRC
2.42	2.86	1.33	6.9	5.2	36.0	12.6	INK-128	mTOR
2.12	2.51	1.48	219.8	148.9	617.6	246.5	PP242	mTOR
2.06	1.93	2.36	124.7	38.6	227.5	59.9	Ponatinib	BCR/ABL/FLT3/RET/KIT/FGFR/PDGFR
2.01	1.69	3.66	1024.3	282.8	3091.3	1480.1	GDC-0941	PI3K
1.98	6.42	0.50	11.5	22.9	48.9	7.6	Elesclomol	Hsp90/apoptosis
1.95	2.13	1.41	135.9	96.5	617.6	289.4	PI-103	P13K
1.91	2.68	1.20	3468.4	2512.9	5889.4	2291.9	Crenolanib	PDGFRA/B
1.86	1.92	1.84	7414.1	4678.4	7245.4	3163.3	Crizotinib	ALK
1.85	11.19	1.38	10000.0	7245.4	4074.8	364.1	pan-JAK	JAK
1.82	1.63	2.74	4678.4	1203.3	3163.3	3236.9	NVP-ADW742	IGF1R
1.78	1.48	2.45	6761.8	2755.2	9121.1	6167.0	Regorafenib	c-KIT/VEGFR1,2/B-Raf/RET/PDGFR
1.77	8.49	1.00	10000.0	10000.0	10000.0	913.0	Ruxolitinib	JAK
1.74	6.76	1.00	10000.0	10000.0	10000.0	1480.1	Tofacitinib	JAK
1.69	2.38	1.32	159.5	121.2	159.5	67.1	BMS-387032	CDK2
1.68	1.35	2.19	1259.9	576.4	1203.3	892.3	XL-880	MET, VEGFR2,KDR
1.66	2.34	1.20	502.2	417.9	661.7	282.8	PKC-412	PKC
1.59	1.00	3.89	10000.0	2571.4	10000.0	10000.0	H-89	PKA
1.56	1.10	2.56	550.5	214.8	502.2	458.1	NVP-TAE-684	ALK
1.53	1.99	1.02	4787.3	4678.4	10000.0	5012.9	PHA-665752	c-Met
1.52	2.65	1.29	150.6	116.5	60.9	23.0	Lestaurtinib	FLT3/JAK2/Trk
1.47	2.14	1.17	4467.8	3802.9	3631.8	1699.2	Cytopia	JAK1/JAK2
1.45	1.29	1.78	2345.2	1319.3	3468.4	2692.5	GSK-1838705A	IGF1R and ALK
1.39	1.99	1.05	235.4	224.9	252.2	126.9	AT-7519	CDK
1.38	1.02	1.86	3163.3	1699.2	2399.8	2345.2	A-674563	AKT
1.32	1.00	1.95	10000.0	5129.6	10000.0	10000.0	Vandetanib	VEGFR, EGFR, RET
1.32	1.02	1.66	10000.0	6026.6	7245.4	7080.5	Nintendanib	VEGFR, PDGFR, FGFR
1.30	1.48	1.05	3389.4	3236.9	6607.9	4467.8	Ruboxistaurin	PKCB
1.30	2.10	0.98	11.5	11.7	9.7	4.6	BI-2536	PLK1
1.27	1.29	1.26	7080.5	5624.4	6310.6	4898.8	ABT-869	VEGFR/PDGFR/KDR/CSF1R
1.27	1.07	1.55	10000.0	6457.5	10000.0	9333.5	MGCD-265	MET/VEGFR/Tie2
1.26	3.15	0.33	3.0	8.9	13.9	4.4	YM-155	survivin
1.26	1.79	1.00	414.8	414.8	356.5	199.6	AKT IV	AKT
1.23	0.93	1.74	10000.0	5755.4	9333.5	10000.0	KI-20227	CSF1R, VEGFR, c-KIT PDGFRB
1.22	0.81	2.19	10000.0	5889.4	10000.0	10000.0	Pazopanib	VEGFR/c-KIT
1.21	0.71	2.40	10000.0	4169.7	6919.3	9773.4	CI-1033	EGFR/ERB2
1.21	2.14	0.78	7763.5	10000.0	10000.0	4678.4	SU11274	MET
1.21	0.93	1.69	10000.0	5496.4	10000.0	10000.0	Sorafenib	VEGFR, PDGFR, RAF
1.20	1.45	0.93	5755.4	6167.0	10000.0	6919.3	Bosutinib	Src, Abl
1.19	1.00	1.66	6607.9	3982.1	10000.0	10000.0	Cabozanitinib	MET and VEGFR2

1.18	1.48	0.96	3802.9	3982.1	4571.9	3091.3	BMS-345541	IKK
1.15	1.00	1.74	4266.8	2455.7	10000.0	10000.0	Cediranib	VEGFR
1.14	1.68	0.45	17.2	38.2	82.3	48.9	BEZ235	PI3K/mTOR
1.13	1.17	1.10	10000.0	9121.1	10000.0	8512.4	AZD-1480	JAK-2
1.13	2.32	0.80	167.0	209.9	139.0	59.9	Flavopiridol	cdk
1.10	1.22	1.00	7.5	7.5	7.9	6.5	Staurosporin	PKC, wide range of targets
1.10	1.70	1.00	3802.9	3802.9	1097.5	646.7	HKI-272= neratinib	ERB2/EGFR
1.09	2.34	0.89	8913.5	10000.0	3716.4	1585.9	EKB-569= pelitinib	ERB1,2,4
1.09	2.14	0.33	2139.0	6457.5	10000.0	4678.4	ABT-737	Bcl-2
1.07	1.29	0.98	6310.6	6457.5	3716.4	2885.0	JNJ-7706621	CDK/cyclin, AuroraA and B
1.07	0.69	1.78	2188.8	1231.3	1585.9	2291.9	Afatinib	ERB2/EGFR
1.05	1.05	1.05	10000.0	9550.9	10000.0	9550.9	PP2	Src family, LCK, FYN, HCK
1.04	2.56	1.00	10000.0	10000.0	692.8	270.2	NF-kB	NF-kB
1.02	1.05	1.00	10000.0	10000.0	10000.0	9550.9	PLX-4720	Raf, BRAFV600E, CRAFY340D/Y341D
1.00	1.15	1.00	10000.0	10000.0	187.2	163.2	Go6976	PKC
1.00	1.00	1.00	10000.0	10000.0	10000.0	10000.0	GSK-690693	AKT
1.00	1.00	1.00	10000.0	10000.0	10000.0	10000.0	PHT-427	AKT, PDPK1
1.00	1.00	1.00	10000.0	10000.0	10000.0	10000.0	SB-431542	ALK5
1.00	1.00	1.00	10000.0	10000.0	10000.0	10000.0	AMPK	AMPK/KDR/VEGFR2/ALK2/BMPR1
1.00	1.00	1.00	10000.0	10000.0	10000.0	10000.0	GDC-0879	B-RAFV600E/pERK
1.00	1.00	1.00	10000.0	10000.0	10000.0	10000.0	Nilotinib	BCR-ABL/KIT/LCK/EPHA/DDR
1.00	1.00	1.00	10000.0	10000.0	10000.0	10000.0	Imatinib	BCR/ABL, KIT
1.00	1.00	1.00	1000.0	1000.0	1000.0	1000.0	Dasatinib	BCR/ABL, SRC, c-Kit
1.00	1.00	1.00	10000.0	10000.0	10000.0	10000.0	JNJ-38877605	c-MET
1.00	1.00	1.00	10000.0	10000.0	10000.0	10000.0	KN92	CAMKII
1.00	1.00	1.00	10000.0	10000.0	10000.0	10000.0	KN93	CAMKII
1.00	1.00	1.00	10000.0	10000.0	10000.0	10000.0	STO609	CAMKK
1.00	1.00	1.00	10000.0	10000.0	10000.0	10000.0	CYC-202	cdc/cdk/cyclin
1.00	1.00	1.00	10000.0	10000.0	10000.0	10000.0	GW-2580	CSF1R
1.00	1.00	1.00	10000.0	10000.0	10000.0	10000.0	JNJ-28312141	CSF1R/FLT3
1.00	1.00	1.00	10000.0	10000.0	10000.0	10000.0	Gefitinib	EGFR
1.00	1.00	1.00	10000.0	10000.0	10000.0	10000.0	Erlotinib	EGFR/JAK2V617F
1.00	1.00	1.00	10000.0	10000.0	10000.0	10000.0	MLN-518= tandutinib	FLT3, PDGFRB, KIT
1.00	1.00	1.00	10000.0	10000.0	10000.0	10000.0	CHIR-99021	GSK3-B
1.00	1.00	1.00	10000.0	10000.0	10000.0	10000.0	vismodegib	hedgehog
1.00	1.00	1.00	10000.0	10000.0	10000.0	10000.0	GSK-1904529A	IGF1R and IR
1.00	1.00	1.00	5000.0	5000.0	5000.0	5000.0	MLN-120B	IKKB
1.00	1.00	1.00	10000.0	10000.0	10000.0	10000.0	AB-1010= masitinib	KIT/PDGFR
1.00	1.00	1.00	10000.0	10000.0	10000.0	10000.0	BIRB-796	MAPK
1.00	1.00	1.00	10000.0	10000.0	10000.0	10000.0	SB-202190	MAPK
1.00	1.00	1.00	10000.0	10000.0	10000.0	10000.0	SB-203580	MAPK
1.00	1.00	1.00	10000.0	10000.0	10000.0	10000.0	SGX-523	MET
1.00	1.00	1.00	10000.0	10000.0	10000.0	10000.0	VX-745	p-38a MAPK
1.00	1.00	1.00	10000.0	10000.0	10000.0	10000.0	p38	p38
1.00	1.00	1.00	1000.0	1000.0	1000.0	1000.0	Sunitinib	PDGFR, VEGFR, KIT, RET, CSF1R, FLT3
1.00	1.00	1.00	10000.0	10000.0	10000.0	10000.0	Cal101	PI3K
1.00	1.00	1.00	10000.0	10000.0	10000.0	10000.0	TG-100-115	PI3K
1.00	1.00	1.00	10000.0	10000.0	10000.0	10000.0	KU-55933	PI3K/mTOR/ATM
1.00	1.00	1.00	10000.0	10000.0	10000.0	10000.0	AZD0530= saracatinib	Src/Fyn/Lyn/Blk/Fgr/Lck
1.00	1.00	1.00	10000.0	10000.0	10000.0	10000.0	S31-201= NSC 74859	STAT3
1.00	1.00	1.00	10000.0	10000.0	10000.0	10000.0	XAV-939	TNKS1, 2 (wnt b-catenin pathway)
1.00	1.00	1.00	10000.0	10000.0	10000.0	10000.0	PTK-787= vatalanib	VEGFR, KIT, PDGFR

1.00	1.00	1.00	10000.0	10000.0	10000.0	10000.0	AMG-706= motesanib	VEGFR1,2,3/PDGFR/cKIT/RET
0.99	1.51	0.38	2139.0	5624.4	10000.0	6607.9	CHIR-258= dovitinib	FLT3/c-KIT/FGFR1/FGFR3/VEGFR1,2,3,/PDGFR/CSF1R
0.95	0.86	1.05	10000.0	9121.1	10000.0	10000.0	Lapatinib	ERB2/EGFR
0.94	1.00	0.88	2239.7	3891.5	10000.0	10000.0	AC-220= quizartinib	FLT3
0.91	9.76	0.00	12.7	10000.0	10000.0	1024.3	GSK-1120212	MEK1/2
0.89	0.43	2.95	6607.9	2239.7	4266.8	10000.0	MK-2206	AKT1,2,3
0.82	0.63	1.00	10000.0	10000.0	6310.6	10000.0	CHIR-265	B-RAF/VEGFR
0.80	0.32	1.15	1048.1	913.0	209.9	661.7	MLN-8054	Aurora A
0.79	0.78	0.81	2345.2	2885.0	3716.4	4787.3	KW-2449	FLT3/ABL/FGFR1/AuroraA
0.73	0.41	2.75	4467.8	1622.8	4074.8	10000.0	AV-951= tivozanib	VEGFR
0.59	0.45	1.20	135.9	113.2	224.9	502.2	17-AAG	Hsp90
0.58	0.81	0.27	2042.7	7586.8	8129.3	10000.0	PD-184352	MEK/MAPK
0.53	1.00	0.07	692.8	10000.0	10000.0	10000.0	AZD-1152= barasertib	Aurora B
0.52	1.00	0.04	447.7	10000.0	10000.0	10000.0	AZD-6244= selumetinib	MEK1
0.48	0.30	1.20	2952.2	2455.7	3021.0	10000.0	PRT062607	Syk
0.47	0.20	2.51	3312.3	1319.3	1996.3	10000.0	Axitinib	VEGFR1,2,3/PDGFR/cKIT
0.40	0.25	0.76	10415.0	13649.8	8164.8	32435.8	JNK II	c-JUN
0.37	0.17	0.41	205.2	502.2	14.5	84.2	MLN-8237= alisertib	Aurora A
0.13	0.25	0.08	115.8	1446.4	170.8	692.8	Bortezomib	proteasome inhibitor
0.05	0.01	0.38	468.7	1231.3	129.8	10000.0	VX-680= Tozasertib	pan-Aurora

Supplementary Figure 1:

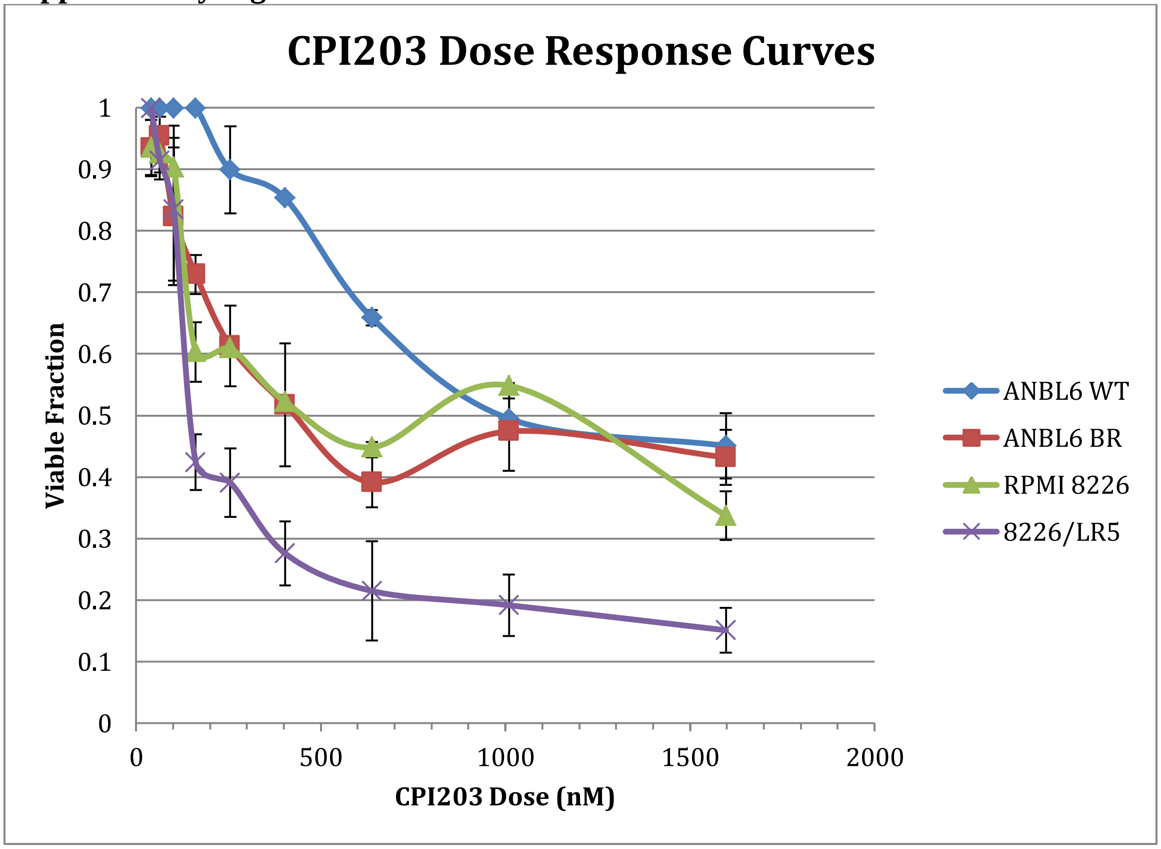

Supplementary Figure 1: CPI203 dose response curves for paired cell lines. Parental (RPMI 8226, ANBL6 WT) and melphalan (LR5/8226) and BTZ (ANBL6 BR) resistant cell lines were treated with single agent CPI203 at various doses. Cell viability at 72 hours was measured using a tetrazolium-based MTS assay.
